# Karyotype stability in the family Issidae (Hemiptera, Auchenorrhyncha) revealed by chromosome techniques and FISH with telomeric (TTAGG)*n* and 18S rDNA probes

**DOI:** 10.3897/CompCytogen.v10i3.9672

**Published:** 2016-08-31

**Authors:** Anna Maryańska-Nadachowska, Boris A. Anokhin, Vladimir M. Gnezdilov, Valentina G. Kuznetsova

**Affiliations:** 1 Institute of Systematics and Evolution of Animals, Polish Academy of Sciences, Sławkowska 17, 30-016 Kraków, Poland; 2 Zoological Institute, Russian Academy of Sciences, Universitetskaya nab. 1, 199034 St. Petersburg, Russia

**Keywords:** Fulgoroidea, Issidae, karyotypes, C-banding, NORs, fluorochrome staining, FISH, (TTAGG)*_n_*, 18S rDNA

## Abstract

We report several chromosomal traits in 11 species from 8 genera of the planthopper family Issidae, the tribes Issini, Parahiraciini and Hemisphaeriini. All species present a 2n = 27, X(0) chromosome complement known to be ancestral for the family. The karyotype is conserved in structure and consists of a pair of very large autosomes; the remaining chromosomes gradually decrease in size and the X chromosome is one of the smallest in the complement. For selected species, analyses based on C-, AgNOR- and CMA_3_-banding techniques were also carried out. By fluorescence *in situ* hybridization, the (TTAGG)*_n_* probe identified telomeres in all species, and the major rDNA loci were detected on the largest pair of autosomes. In most species, ribosomal loci were found in an interstitial position while in two species they were located in telomeric regions suggesting that chromosomal rearrangements involving the rDNA segments occurred in the evolution of the family Issidae. Furthermore, for 8 species the number of testicular follicles is provided for the first time.

## Introduction

During the last decades, the worldwide planthopper family Issidae was comprehensively revised based on morphological features (Emeljanov 1999, Gnezdilov 2003a, b, 2007, 2012a, b, 2013a, b, c, Gnezdilov and Wilson 2006, Gnezdilov et al. 2014). Several groups treated previously as Issidae subfamilies were upgraded to the family rank (Caliscelidae and Acanaloniidae). The subfamilies Trienopinae and Tonginae were transferred as tribes to the families Tropiduchidae and Nogodinidae respectively, while the tribes Adenissini and Colpopterini were transferred to the Caliscelidae and Nogodinidae, respectively. The term “issidoid group” has been suggested for grouping the families Issidae, Caliscelidae, Acanaloniidae, Tropiduchidae and Nogodinidae (Gnezdilov 2013b, Gnezdilov et al. 2015).

As a result of these changes, the family Issidae *sensu stricto* is now considered to comprise more than 1000 species and subspecies with around 170 genera classified within the only nominatypical subfamily Issinae, including three tribes, Issini Spinola, 1839, Hemisphaeriini Melichar, 1906 and Parahiraciini Cheng & Yang, 1991 (Gnezdilov 2013a, Bourgoin 2016). The largest tribe Issini exhibits worldwide distribution while the two other tribes are mainly endemics of the Oriental Region (Gnezdilov 2013a, Gnezdilov et al. 2014).

Recent molecular data on the Issidae *sensu lato* using a partial sequence of the 18S rDNA and the *wingless* gene (Sun et al. 2015) are not congruent in all cases with the above classification resulted from morphological data. However, the monophyly of the Issidae *s. str.* and the existence of three distinct phylogenetic lineages (tribes) were confirmed. The phylogenetic position of another tribe, the Tongini, might be an artifact (Gnezdilov et al. 2015). Thus in our current paper we follow the morphology-based classification.

Up to now, studies on the Issidae *s. str.* karyotypes were performed on 36 species (20 genera), all being from the tribe Issini (Maryańska-Nadachowska et al. 2006, Kuznetsova et al. 2010). Pioneering karyological studies (Parida and Dalua 1981, Tian et al. 2004) and later comparisons based on standard (Schiff-Giemsa) and differential (Ag-NOR and DAPI/CMA_3_) staining techniques (Maryańska-Nadachowska et al. 2006, Kuznetsova et al. 2009, 2010) showed that issids are characterized by a pronounced karyotypic conservatism. They have strikingly similar karyotypes with only three male diploid chromosome numbers: 27, 26 and 25. The most common karyotype of 2n = 27 (26 + X) is considered as phylogenetically ancestral in the family (Kuznetsova et al. 2010) and appears similar in structure among the species studied. It consists of a pair of very large autosomes; the remaining chromosomes gradually decrease in size, and the X chromosome is among the small chromosomes of the set. As revealed by CMA_3_ staining and silver nitrate impregnation (AgNOR staining), the largest autosomal pair bears nucleolus organizer regions (NORs) in all studied species. In contrast to the above chromosome techniques, C-banding revealed differences between species in the amount and distribution of heterochromatin, and its staining affinity using DAPI and CMA_3_ (Kuznetsova et al. 2009, 2010). Thus, despite the vast variation within the Issidae, the cytogenetics of this group remains poorly explored and no molecular cytogenetic techniques have previously been applied.

Recent publications dealing with karyotypes of the Issidae have additionally reported some data on internal reproductive organs, mainly on the number of testicular follicles (Maryańska-Nadachowska et al. 2006, Kuznetsova et al. 2010). Issids were shown to be characterized by testes with rather numerous follicles, ranging from 4 (*Palmallorcus punctulatus*) to 30 (*Zopherisca tendinosa*) per testis, with a predominant number of 10.

In this paper we report karyotypes of 11 species in 8 genera of the tribes Issini, Parahiraciini and Hemisphaeriini, studied by several chromosome techniques, including fluorescence *in situ* hybridization (FISH) with (TTAGG)*_n_* telomeric and 18S rDNA probes. We particularly focused on whether karyotypes with the same chromosome number show different patterns if new molecular cytogenetic markers are applied. In addition, we present, for the first time, the number of testicular follicles for 8 species, including first observations on members of the tribes Parahiraciini and Hemisphaeriini. All currently available data on the family Issidae are summarized and tabulated.

## Material and methods

Details on the material analyzed, including the geographical location, number of specimens, information about the authorship of the noted specific names, diploid (2n) chromosome number, sex-determining mechanism in males, cytogenetic methods used in karyotyping and the number of testicular follicles are given in Table 1. Moreover, Table 1 summarizes all species studied so far in respect to karyotype and reproductive system in the family Issidae.

**Table 1.** List of the Issidae species studied in respect to karyotype and testis structure^1^

Taxa	Collection locality	No. of males (m) and females (f) studied	2n♂	Number of follicles	Method	Gaps/AgNORs/rDNA FISH location on the largest pair of autosomes	Source
**Issidae Spinola** **Issinae Spinola** **Issini Spinola**							
*Agalmatium bilobum* (Fieber, 1877)	Russia, Greece Italy, Gemona del Friuli, Alps, ca. 25 km north from Udine, Udine prov., 07.07.2005, leg. A. Maryańska-Nadachowska Bulgaria, Krupnik, S from Simitli, Struma River valley, 9.05.2007, leg. A. Maryańska-Nadachowska	? m, ? f 3m 1f 2m	- 26+X -“-	11/11 8/8 11/11 8/8 11/11	- Schiff rDNA FISH	- Interstitial gap Interstitial cluster	Emeljanov and Kuznetsova 1983 Maryańska-Nadachowska et al. 2006 Present data **Fig. 11**
*Agalmatium flavescens* (Olivier, 1791)	Spain, Sierra d’Alhamilla, Almeria prov., 3.06.2006, leg. A. Maryańska-Nadachowska -“-	2m 1m	26+X -“-	11/11 -“-	Schiff rDNA FISH	- Interstitial cluster	Maryańska-Nadachowska et al. 2006 Present data **Fig. 12**
*Bergevinium ?malagense* (Matsumura, 1910)	Spain, El Burgo, Malaga prov., 11.06.2005, leg. A. Maryańska-Nadachowska	2m	26+X	9/9	Schiff	-	Maryańska-Nadachowska et al. 2006
*Brahmaloka* sp.	India	?m	24+X	-	-	-	Parida and Dalua 1981
*Bubastia obsoleta* (Fieber, 1877)	Greece, Litohoro, eastern slopes of Mt. Olympus, Pieria District, 17.05.2007, leg. A. Maryańska-Nadachowska	4m	26+X	10/10	Schiff, C-banding, DAPI	-	Kuznetsova et al. 2010
*Bubastia saskia* Dlabola, 1984	Greece, Varvara, Stratoniko Range (600-800 m a.s.l.), Halkidiki District, 11.06.2007, leg. A. Maryańska-Nadachowska	4m	26+X	10/10	Schiff, C-banding, DAPI	-	Kuznetsova et al. 2010
*Bubastia taurica* (Kusnezov, 1926)	Russia, Krasnodar Territory, near Gelendzhik, 30.08.2004, leg. V. Gnezdilov	1m	26+X	10/10	Schiff	-	Maryańska-Nadachowska et al. 2006
*Conosimus coelatus* Mulsant & Rey, 1855	France, prov. Vaison-la-Romaine, 1.06.2010, leg. A. Maryańska-Nadachowska	2m	26+X	9/9	Schiff, AgNOR	Interstitial gap Interstitial NOR	Present data **Fig. 1a, b**
*Corymbius tekirdagicus* (Dlabola, 1982)	Greece, Litohoro eastern slopes of Mt. Olympus, Pieria District, 17.05.2007, leg. A. Maryańska-Nadachowska	2m	26+X	10/10	Schiff	-	Kuznetsova et al. 2010 as: *Kervillea tekirdagica*
*Dentatissus damnosus* (Chou & Lu, 1985)	China -“-	?m ?m ?f	26+X -	- 18/18 9/9	Phenol fuchsine	- -	Tian et al. 2004 as *Sivaloka damnosa* Meng et al. 2010 as *Sivaloka damnosa*
*Falcidius doriae* (Ferrari, 1884)	Italy, Caltabellotta, alt. ca. 900 m a.s.l., ca. 30 km north from Sciacca, southern Sicily, 22.05.2006, leg. A. Maryańska-Nadachowska	3m	26+X	10/10	C-banding	-	Kuznetsova et al. 2010
*Falcidius limbatus* (A. Costa, 1864)	Italy, Chiaramonte, ca. 15 km north from Ragusa, southern Sicily, 16.05.2006, leg. A. Maryańska-Nadachowska	4m	24+XY	-	C-banding	Interstitial gap	Kuznetsova et al. 2010
*Hysteropterum albaceticum* Dlabola, 1983	Spain, Soria prov., 07.2005, leg. A. Maryańska-Nadachowska	3m	26+X	10/10	Schiff	-	Maryańska-Nadachowska et al. 2006
*Hysteropterum dolichotum* Gnezdilov & Mazzoni, 2004	Spain, Segovia prov., 07.2005, leg. A. Maryańska-Nadachowska	2m	26+X	-	Schiff	-	Maryańska-Nadachowska et al. 2006
*Hysteropterum vasconicum* Gnezdilov, 2003	Spain, Soria prov., 07.2005, leg. A. Maryańska-Nadachowska	3m	26+X	10/10	Schiff	-	Maryańska-Nadachowska et al. 2006
*Issus coleoptratus* (Fabricius, 1781)	Spain, near Almonte, 26.06.2004 (south Spain), leg. A. Maryańska-Nadachowska	2m	26+X	13/13	Schiff	-	Maryańska-Nadachowska et al., 2006
*Issus lauri* Ahrens, 1814	Italy (Sicily), Acireale, east Sicily, 2.06.2006, leg. A. Maryańska-Nadachowska -“-	2m 1f 1m	26+X - -“-	13/13 13/12 13/13	Schiff rDNA FISH	Terminal gap Terminal cluster	Maryańska-Nadachowska et al. 2006 Present data **Fig. 13**
*Kervillea basiniger* (Dlabola, 1982)	Greece, Litohoro, eastern slopes of Mt. Olympus, Pieria District, 17.05.2007, leg. A. Maryańska-Nadachowska -“-	2m 1m	26+X -“-	10/10 -“-	Schiff rDNA FISH	- Interstitial cluster	Kuznetsova et al. 2010 Present data **Fig. 14**
*Kervillea scoleogramma* (Fieber, 1877)	Turkey, Gülcük, (1100m a.s.l., Boz Dağlar ca. 18 km north from Edemis, prov. Izmir, 3.06.2010, leg. A. Maryańska-Nadachowska	3m	26+X	12/12	Schiff	Interstitial gap	Present data **Fig. 2 a, b**
*Latematium latifrons* (Fieber, 1877)	Bulgaria, Central Rodopy Mts., 2010, leg. A. Maryańska-Nadachowska	3m	26+X	12/12	Schiff	-	Present data **Fig. 3**
*Latilica maculipes* (Melichar, 1906)	Italy, Gemona del Friuli, Alps, ca. 25 km north from Udine, Udine prov., 07.07.2005, leg. A. Maryańska-Nadachowska	2m	24+X	10/10	Schiff	-	Maryańska-Nadachowska et al. 2006
*Latissus dilatatus* (Fourcroy, 1785)	Italy, Lagonegro, ca.15 km north from Lauria, 11.06.2006, leg. A. Maryańska-Nadachowska	5m	26+X	12/12	Schiff, C-banding, AgNOR, DAPI	Subtelomeric gaps, NORs	Kuznetsova et al. 2010
*Mulsantereum abruzicum* (Dlabola, 1983)	Italy, Sicily, Nébrodi Mountains, western part, surroundings of di Luminaria (1260 m), dell Obolo Pass (1503 m), 27.05.2006, leg. A. Maryańska-Nadachowska	2m	26+X	10/10	Schiff	-	Kuznetsova et al. 2010
Mycterodus (Mycterodus) drosopoulosi Dlabola, 1982	Greece, near Athens, Parnitha Mt., 05.05.2015, leg. V. Gnezdilov	2m 1f	26+X -	10/13, 7/18 15/15	Schiff	-	Present data **Fig. 4**
Mycterodus (Mycterodus) etruscus Dlabola, 1980	Italy, Passo del Muraglione, 907 m a.s.l., ca. 50 km north-east from Firenze, 14.06.2006, leg. A. Maryańska-Nadachowska	1m	26+X	16/16	Schiff	-	Kuznetsova et al. 2010
Mycterodus (Mycterodus) intricatus Stål, 1861	Crimea, Chatyr-Dag, 1000 m a.s.l., 06.2008, leg. A. Maryańska-Nadachowska	1m	26+X	20/20	Schiff, C-banding	-	Kuznetsova et al. 2010
Mycterodus (Semirodus) colossicus (Dlabola, 1987)	Greece, Varvara, Stratoniko Range (600-800 m a.s.l), Halkidiki District, 11.06.2007, leg. A. Maryańska-Nadachowska	3m	26+X	18/18	Schiff, C-banding, AgNOR, DAPI	Interstitial gaps	Kuznetsova et al. 2010
Mycterodus (Semirodus) pallens (Stål, 1861)	Greece, leg. S. Drosopoulos -“-	1m 1f 1m	26+X - -“-	18/18 9/9 -“-	Schiff rDNA FISH	- Interstitial (?) clusters	Maryańska-Nadachowska et al. 2006 Present data **Fig. 15**
Mycterodus (Semirodus) sp.	Turkey, Kayacidad Mts (700-800 m a.s.l), south from Canakale, 06.2010, leg. A. Maryańska-Nadachowska	2m	26+X	-	Schiff, C-banding	-	Present data **Fig. 5a, b**
*Palaeolithium distinguendum* (Kirschbaum, 1868)	Spain, Goñar, Almeria prov., 07.2005, leg. A. Maryańska-Nadachowska -“-	5m 1m	26+X -“-	7/13, 8/8, 8/11 9/9, 9/11 8/8	Schiff rDNA FISH	- Interstitial clusters	Maryańska-Nadachowska et al. 2006 Present data **Fig. 16**
*Palmallorcus balearicus* (Dlabola, 1982)	Spain, Mazagón, Huelva prov, 14.06.2005, leg. A. Maryańska-Nadachowska	3m	26+X	9/10, 10/10, 11/11	Schiff	-	Maryańska-Nadachowska et al. 2006
*Palmallorcus nevadensis* (Linnavuori, 1957)	Spain, Sierra de la Nieves, Malaga prov., 4.06.2005, leg. A. Maryańska-Nadachowska	2m	26+X	10/10	Schiff	Interstitial gaps	Maryańska-Nadachowska et al. 2006
*Palmallorcus punctulatus* (Rumbur, 1840)	Spain, Avila prov., 07.2005, leg. A. Maryańska-Nadachowska	1m	26+X	?4/4	Schiff	Interstitial gaps	Maryańska-Nadachowska et al. 2006
*Sarnus* sp.	Chile, La Campana, 2014, leg. A. Emeljanov	4m	26+X	6/6	Schiff, AgNOR,	? ?	Present data **Fig. 6a, b**
*Scorlupaster asiaticum* (Lethierry, 1878)	Kazakhstan, 42°50´20.724´´N 71°10´12.900´´E, 29.07.2006, leg. V.Gnezdilov	2m	26+X	9/9	Schiff	-	Kuznetsova et al. 2010
*Scorlupella discolor* (Germar, 1821)	Crimea, Chatyr-Dag, 1000 m a.s.l., 06.2008, leg. A. Maryańska-Nadachowska -“-	1m 1m	26+X -“-	6/6 -“-	Schiff rDNA FISH	- Interstitial ? clusters	Kuznetsova et al. 2010 Present data **Fig. 17**
*Thionia obtusa* Melichar, 1906	Southern Mexico, 11. 2012, leg. A. Maryańska-Nadachowska,	1m	26+X	-	Schiff	Interstitial gaps	Present data **Fig. 7**
*Tingissus tangirus* (Matsumura, 1910)	Spain, El Burgo, Malaga prov. 20.06.2006, leg. A. Maryańska-Nadachowska	4m 1f	26+X -	10/10 6/6	Schiff	-	Maryańska-Nadachowska et al. 2006
*Tshurtshurnella pythia* Dlabola, 1979	Greece, 2003, leg. S. Drosopoulos	3m 1f	26+X	12/12 7/7	Schiff	-	Maryańska-Nadachowska et al. 2006
*Zopherisca penelopae* (Dlabola, 1974)	Greece, 2003, leg. S. Drosopoulos Greece, Myrsini, ca 20 km W from Githio, Lakonia distr., Peloponessus, 2007.05. 24, leg. A. Maryańska-Nadachowska	3m 1m	26+X -“-	24/24 -“-	Schiff rDNA FISH	- Interstitial clusters	Maryańska-Nadachowska et al. 2006 Present data **Fig. 19**
*Zopherisca skaloula* Gnezdilov & Drosopoulos, 2006	Greece, Skaloula village, 2003, leg. S. Drosopoulos	1m	26+X	30/30	Schiff	-	Maryańska-Nadachowska et al. 2006
*Zopherisca tendinosa* (Spinola, 1839)	Greece, Achladokambos, ca. 20 km E from Tripoli, Arkadia District, Peloponessus, 23.05.2007, leg. A. Maryańska-Nadachowska -“-	3m 1m	26+X -“-	28/28 -“-	Schiff, C-banding, DAPI rDNA FISH	- Terminal clusters	Kuznetsova et al. 2010 Present data **Fig. 20**
**Parahiraciini**							
*Thabena* sp.	Vietnam, Dak Lak Prov, Yok Don Nat. Park, 20.06.2014. leg. V. Gnezdilov	1m	26+X	11/11	Schiff, DAPI/CMA_3_ rDNA FISH	Interstitial clusters	Present data **Fig. 8a, b** **Fig. 18**
**Hemisphaeriini**							
*Hemisphaerius interclusus* Noualhier, 1896	South Vietnam, Cat Tien, Nat. Res., 2012, leg. V. M. Gnezdilov	2m	26+X	8/11, 12/12	Schiff	Interstitial gaps	Present data **Fig. 9**
*Hemisphaerius* sp.	Indonesia, 2011, leg. D.A. Gapon	4m	26+X	8/9, 11/11, 12/12, 12/12	Schiff rDNA FISH	- Interstitial clusters	Present data **Figs 10, 21a, b**

1 With several exceptions (Tian et al. 2004, Meng et al. 2010, Parida and Dalua 1981), all species were identified by V.M. Gnezdilov.

### Insects

All specimens were identified by V.M. Gnezdilov. Several species were identified only to the genus level because of taxonomic difficulties in these genera. Only males were used for chromosome analyses. In the field, males were collected with an insect net, fixed alive in 3:1 fixative (96% ethanol: glacial acetic acid) and stored at +4 °C.

### Slide preparation

Gonads of adult males were used for chromosome analysis. Testes were dissected in a drop of 45% acetic acid and squashed. The coverslips were removed using dry ice. Prior to staining, the preparations were examined by phase contrast microscopy.

### Conventional chromosome staining methods

All the conventional staining techniques used herein were described in detail by Kuznetsova et al. (2009, 2010) for other issid species, i.e., Schiff-Giemsa staining, C-banding, AgNOR-banding and CMA_3_-banding. All species were studied using the standard Schiff-Giemsa technique by Grozeva and Nokkala (1996), whereas the other techniques were used only for selected species (Table 1).

Chromosome banding techniques contribute to the identification of specific chromosomes within karyotypes. AgNOR-banding reveals chromosomal nucleolus organizing regions (NORs) representing the sites for the tandemly arranged 18S and 28S ribosomal RNA genes. The AgNOR-banding presumably differentiates only those NORs which were metabolically active during the preceding interphase (Howell and Black 1982). Some chromosome banding techniques, including C-banding and fluorochrome banding, are strongly dependent on the amount of heterochromatin and its distribution in chromosomes. Chromomycin A_3_ (CMA_3_) reveals the presence of GC-rich heterochromatin, which is usually associated with NOR regions, and thus differentiates NORs regardless of their prior metabolic activity.

### Fluorescence *in situ* hybridization (FISH)

This method was applied for the first time in the family Issidae. We used FISH with a (TTAGG)*_n_* and 18S rDNA probes in 11 species from 8 genera; 9 species from Issini tribe while that one species of the Parahiraciini and Hemisphaeriini tribes (Table 1). FISH with both probes was applied as previously reported (Maryańska-Nadachowska et al. 2013, Golub et al. 2014, Kuznetsova et al. 2015b, c). In brief, chromosome preparations were treated with 100 µg/ml RNase A, and 5 mg/ml Pepsin solution was used to remove excess RNA and proteins. Chromosomes were denatured on a slide in a hybridization mixture with biotinylated 18S rDNA probe from the genomic DNA of *Pyrrhocoris apterus* (Linnaeus, 1758) and rhodaminated (TTAGG)*_n_* probe with addition of salmon sperm DNA and then hybridized for 36 h. Hybridization signals were detected with NeutrAvidin-FITC.

Chromosomes were mounted in antifade medium (ProLong Gold antifade reagent with DAPI; Invitrogen) and covered with a glass coverslip. Chromosome slides were analyzed under a Leica DM 6000 B microscope. Images were taken with a Leica DFC 345 FX camera using Leica Application Suite 3.7 software with an Image Overlay module.

## Results

### Testicular and ovarian follicles

The testicular follicles were counted in 8 species (Table 1). The follicles were tubular and their number ranged from 6 to 18 among the species studied (here and elsewhere numbers are given per testis) and occasionally varied among males and in different testes of the same male, e.g. in *Mycterodus drosopoulosi* (2 males: 10/13, 17/18), *Hemisphaerius* sp. (2 males: 11/8, 12/12) and *Hemisphaerius interclusus* (4 males: 9/8, 11/11, 12/12, 12/12). In the only studied female of *Mycterodus drosopoulosi*, about 15 ovarian follicles were counted in each gonad.

### Conventional and differential chromosome techniques

**Figures 1–8.**
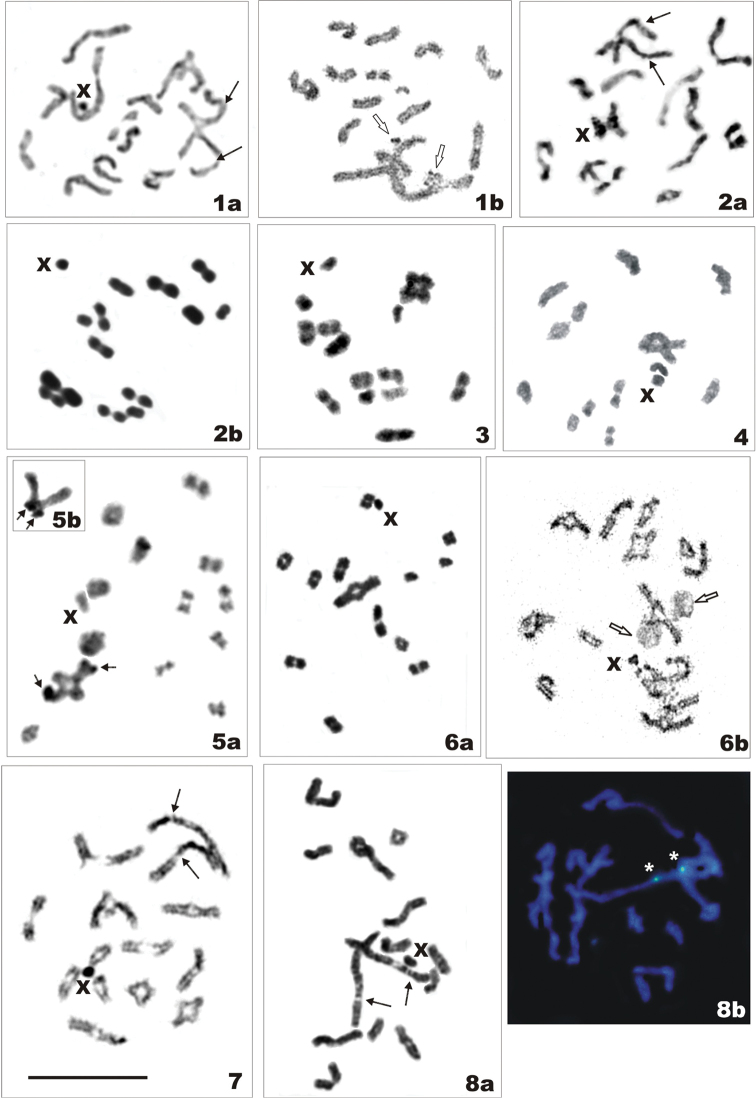
Meiotic analyses of species of the tribes Issini (Figures 1–7) and Parahiraciini (Figure 8), (n = 13 bivalents + X) with different cytogenetic techniques. **1** *Conosimus coelatus*, **a** diakinesis with standard and **b** AgNOR-staining. Arrows point to “secondary” constrictions (gaps) (**a**) and empty arrows NORs (**b**) point to the largest autosomal pair **2** *Kervillea scoleogramma*, **a** diakinesis and **b** metaphase I with standard staining. Arrows point to "secondary" constrictions on the largest autosomal pair (**a**) **3** *Latematium latifrons*, metaphase I with standard staining **4** Mycterodus (Mycterodus) drosopoulosi, diakinesis with standard staining **5** Mycterodus (Semirodus) sp., diakinesis with C-banding. **a** Arrows point to C-bands on the largest and medium-sized bivalents. In the largest bivalent, C-bands are located at the terminal or **b** at the proximal (chiasmate) parts of chromosomes. Short arrows point to C-bands **6** *Sarnus* sp., **a** metaphase I with standard staining and **b** diakinesis with AgNOR-banding. Arrows point to NORs on the largest autosomal pair **7** *Thionia obtusa*, diakinesis with standard staining. Arrows point to “secondary” constrictions on the largest autosomal pair **8** *Thabena* sp. **a** diakinesis with standard staining, and **b** diplotene with CMA_3_-banding. Arrows point to “secondary” constrictions (**a**) and asterisks mark CMA_3_-positive, GC-rich regions (**b**) of the largest autosomal pair. Scale bar =10 µm.

**Figures 9–10.**
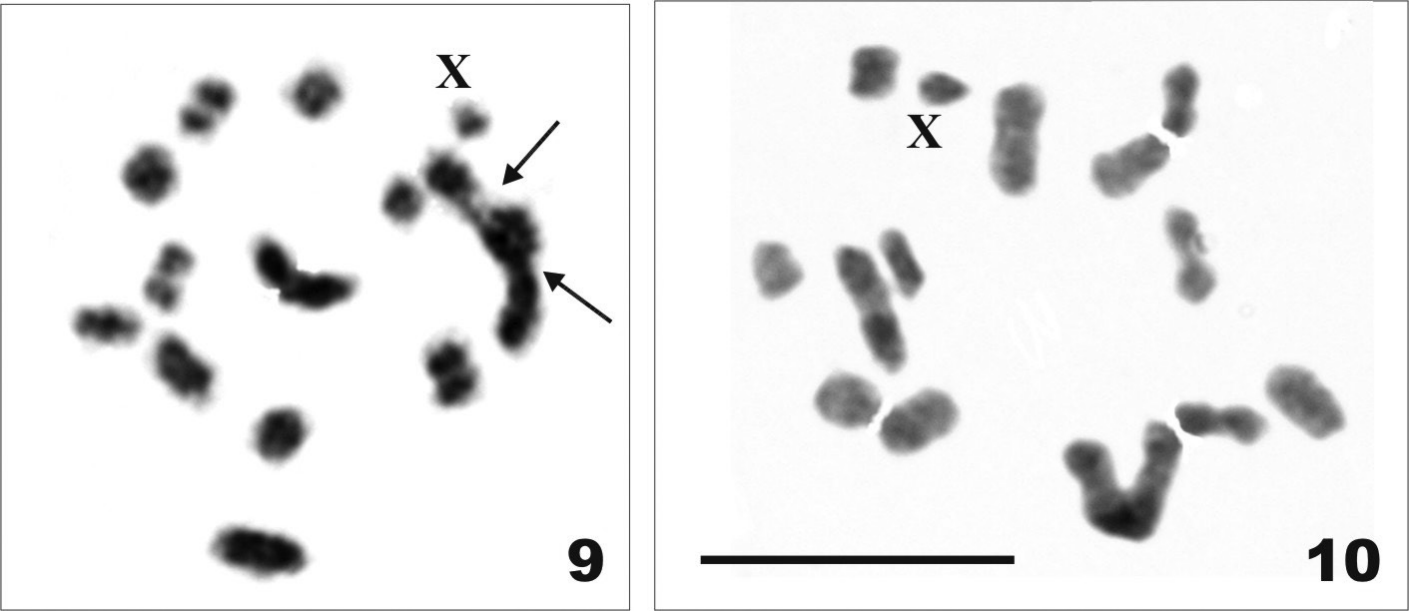
Conventionally stained meiotic karyotypes of two species of the tribe Hemisphaeriini (n = 13 bivalents + X). **9** *Hemisphaerius interclusus*, metaphase I with standard staining. Arrows point to “secondary” constrictions in the largest autosomal pair **10** *Hemisphaerius* sp., metaphase I with standard staining. Scale bar = 10 µm.

Chromosome data on 10 species from 8 genera were obtained for the first time, including first observations on members of the tribes Parahiraciini and Hemisphaeriini (Table 1). Representative photographs of standard and sometimes also differentially stained meiotic karyotypes are presented in Figs 1–10. All species showed holokinetic chromosomes and the same chromosome number in males. In meiotic cells (diakinesis, metaphase I), there were 13 autosomal bivalents and a univalent X chromosome, i.e., 2n = 26 + X. Also, the karyotype structure seemed to be uniform with a pair of very large autosomes, 12 bivalents more or less gradually decreasing in size and the X chromosome as one of the smaller chromosomes of the set. The largest bivalent had a very large “secondary” constriction (a gap) in each homologue (Figs 1a, 2a, 7, 8a, 9). This constriction divided the chromosome into two unequal parts, however it was not always visible, especially when the chromosomes were more condensed (Figs 2b, 3, 4, 5a, 6a, 10). The silver staining technique used in *Conosimus coelatus* and *Sarnus* sp. produced a precipitation of silver at these regions suggesting that they harbor NORs (Figs 1b, 6b). In *Thabena* sp., the CMA_3_/DAPI staining showed homogeneous DAPI staining (results not shown) and distinct patterns of GC-rich blocks (CMA_3_-positive) in the NORs (Fig. 8b). The C-banded karyotype of *Mycterodus* sp. showed prominent telomeric C-bands in the largest and one of the medium-sized bivalents (Fig. 5a, b).

### Fluorescence *in situ* hybridization (FISH)

#### Detection of a tandem telomeric repeat sequence by FISH with a (TTAGG)_n_ probe.

The telomeric probe identified (TTAGG)*_n_* repeats on the chromosomal ends in the nine species analyzed (Table 1), but not all telomeres were distinctly labeled in each chromosome spread (Figs 11–21). Some chromosomes showed only faint hybridization signals.

**Figures 11–18.**
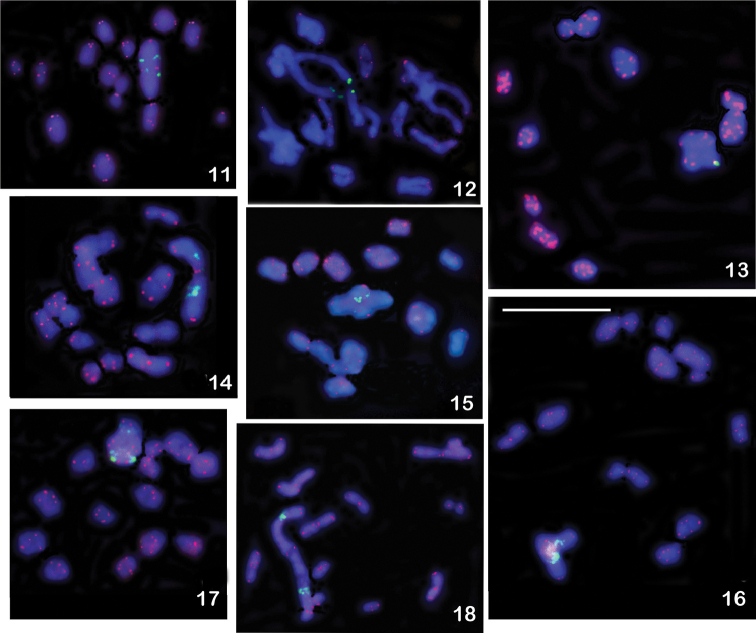
FISH with rDNA (green signals) and telomeric (TTAGG)*_n_* (red signals) probes on male meiotic karyotypes of eleven Issidae species (n = 13 bivalents + X). The rDNA clusters are seen on the largest autosomal pair, located interstitially in all species with the exception of *Issus lauri* (Figure 13) and *Zopherisca tendinosa* (Figure 20) with the terminal location of these clusters. **11** *Agalmatium bilobum*, metaphase I **12** *Agalmatium flavescens*, diplotene-diakinesis transition **13** *Issus lauri*, metaphase I **14** *Kervillea basinger*, metaphase I **15** Mycterodus (Semirodus) pallens, metaphase I **16** *Palaeolithium distinguendum*, metaphase I **17** *Scorlupella discolor*, metaphase I **18** *Thabena* sp., metaphase I. Scale bar = 10 µm.

**Figures 19–21.**
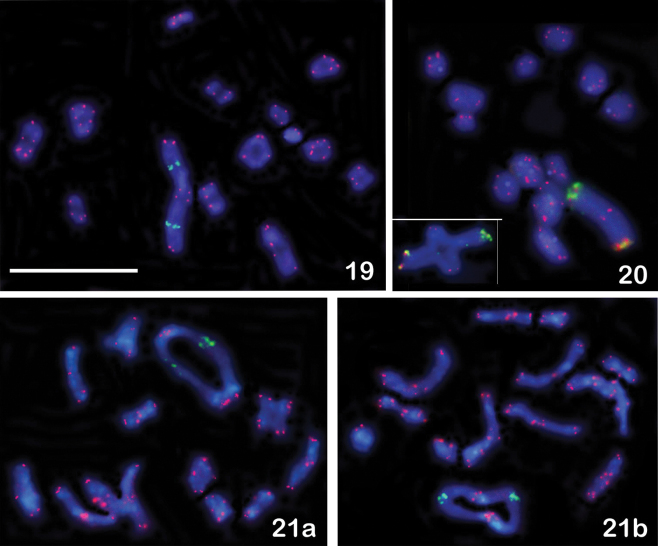
FISH with rDNA (green signals) and telomeric (TTAGG)*_n_* (red signals) probes on male meiotic karyotypes of eleven Issidae species (n = 13 bivalents + X). The rDNA clusters are seen on the largest autosomal pair, located interstitially in all species with the exception of *Issus lauri* (Figure 13) and *Zopherisca tendinosa* (Figure 20) with the terminal location of these clusters. **19** *Zopherisca penelopae*, diakinesis-metaphase I transition **20** *Zopherisca tendinosa*, metaphase I **21** *Hemisphaerius* sp., **a** and **b** diakinesis. Scale bar = 10 µm.

#### Detection of ribosomal genes revealed by FISH with an 18S rDNA probe

In all species, the major rDNA loci were located in the largest autosomal pair. In the majority of species, the rDNA clusters were found in the interstitial position; however in *Issus lauri* and *Zopherisca tendinosa* they were clearly seen in the terminal regions (Figs 13, 20). In some species, rDNA FISH revealed heteromorphism in size of rDNA clusters (Figs 14, 13, 20).

#### Compilation of data on karyotypes and testis structure

We made a thorough compilation of all data reported so far in the family Issidae, including the tribes Issini, Parahiraciini and Hemisphaeriini. Table 1 covers information on a total of 44 species from 27 genera studied in respect to karyotypes and on 40 species from 26 genera studied in respect to the number of testicular follicles.

## Discussion

### Follicle number

The number of testicular follicles per testis, counted here in males of eight species, ranged from 6 to 30, being the lowest in *Sarnus* sp. and the highest in *Zopherisca tendinosa* (both from Issini). In some species, the number of follicles varies among males of the same species and between testes of the same male. Specifically, variation was observed in *Mycterodus drosopoulosi* in which three examined males had testes with 17 and 18; 10 and 13; and 15 and 15 follicles, respectively. As in other planthopper families, in Issidae testicular follicles are of tubular shape. D’Urso et al. (2005) pointed out that Fulgoromorpha are differentiated by this pattern from Cicadomorpha in which follicles are lobular.

The evolutionary trends and the phylogenetic importance of the number of follicles in Auchenorrhyncha were repeatedly discussed in the literature (e.g., Emeljanov and Kuznetsova 1983, Kirillova 1989, D’Urso et al. 2005, Kuznetsova et al. 2009, Gnezdilov 2013b). In some groups variation in this character agrees with their taxonomy and phylogeny. For instance, the number of follicles is conserved at the level of tribes and/or subfamilies within the planthopper families Delphacidae and Dictyopharidae, with changes in this pattern correlated with their overall morphological evolution (Kirillova 1989, Kuznetsova et al. 2009). However, studies of testis structure in the Issidae documented the lability of the follicle number (Maryańska-Nadachowska et al. 2006, Kuznetsova et al. 2010). In 40 species studied so far, a wide range of follicle numbers have been reported, from four (in *Palmallorcus punctulatus*; but *Palmallorcus balearicus* and *Palmallorcus nevadense* have higher numbers, 10 or 11) and six (in *Scorlupella discolor* and *Sarnus* sp.) to 30 (in *Zopherisca skaloula*). Noteworthy are the unusually high numbers (24, 28 and 30) found in the three studied species of the genus *Zopherisca* Emeljanov, 2001. Interestingly, the number of follicles varies between closely related species (in the genera *Kervillea* Bergevin, 1918, *Mycterodus* Spinola, 1839, *Palmallorcus* Gnezdilov, 2003, *Zopherisca*) and even within the same species (e.g. *Palaeolithium distinguendum*, *Palmallorcus balearicus* and *Mycterodus drosopoulosi*) suggesting that evolutionary changes in the follicle number can be relatively rapid in the Issidae. In the opinion of Gnezdilov (2013b), the polymerization of seminal follicles inherent in the Issidae and also in other higher fulgoroid families, such as Nogodinidae, Ricaniidae and Flatidae (Kuznetsova et al. 1998), indicates that these families are relatively young in terms of evolution and that the testis structure has not yet been stabilized within their supraspecific taxa.

Although numbers between 9 and 18 and especially 10 (observed in one third of the species) seem to be more typical for the Issidae, there is still no conclusive evidence of the most characteristic number in this group. This problem can be resolved primarily through improved taxon sampling.

### Karyotypes

The nine species of the Issidae studied here for the first time have broadly similar karyotypes having the male diploid number (2n) of 27 chromosomes, including 13 autosomal pairs and an X(0) sex determination system. The karyotype includes a relatively small X chromosome, one pair of very long autosomes and the remaining autosomes which gradually decrease in size. Issidae, like other Auchenorrhyncha and Hemiptera, have holokinetic chromosomes. The largest bivalent is always NOR-bearing, and NORs are interstitial in the majority of species. The exceptions are *Issus lauri* and *Zopherisca tendinosa*, in which the 18S rDNA cluster is located terminally; this particular pattern probably resulted from inversions. GC-rich DNA segments labeled by CMA_3_ are associated with nucleolus organizer regions.

Our study confirms that Issidae are a group characterized by the high karyotypic conservatism, with the basic karyotype of 2n = 27 (26 + X) (Maryańska-Nadachowska et al. 2006, Kuznetsova et al. 2010). At present, data on karyotypes are available for 44 species (around 4.5 % of the described species) and 27 genera (around 16 % of the recognized genera) in the three currently accepted tribes, Issini, Parahiraciini and Hemisphaeriini. With the exception of *Latilica maculipes* and *Brahmaloka* sp., both with 2n = 24 + X, and *Falcidius limbatus* with 2n = 24 + XY (the Issini), all species have 2n = 27 (26 + X). This makes the monophyletic origin of the latter karyotype an attractive hypothesis and, indeed, the ancestrality of this pattern has been inferred (Maryańska-Nadachowska et al. 2006, Kuznetsova et al. 2010). Every other karyotype could thus have arisen by a single tandem fusion, either between two pairs of autosomes (*Latilica maculipes* and *Brahmaloka* sp., 2n = 24 + X) or between an autosome and the X chromosome (*Falcidius limbatus*, 2n = 24 + XY), respectively. Thus, the chromosome number decreased at least three times in the evolution of the family Issidae. Sex chromosomes of *Falcidius limbatus* are most likely of the neo-XY type. Notably, neo-sex chromosome systems derived via autosome-sex chromosome fusion have been frequently reported in Auchenorrhyncha (see Kuznetsova and Aguin-Pombo 2015). This mechanism, necessarily resulting in reduced chromosome numbers, was clearly involved in sex chromosome diversification of the genus *Falcidius* Stål, 1866, in which the other studied species, *Falcidius doriae*, has the basic chromosome complement of 2n = 27 (26 + X).

The basic karyotype appears conservative in structure within the Issidae, at least as regards the very large pair of autosomes, present in all the studied species. Based on a variety of observations (Giemsa-negative “secondary” constrictions, CMA_3_, AgNOR and rDNA FISH patterns), the largest chromosomes are the NOR-bearing pair in the issid karyotypes.

C- banding has revealed unsuspected patterns of variation in the amount and distribution of constitutive heterochromatin in auchenorrhynchan karyotypes (see Kuznetsova and Aguin-Pombo 2015), and this is also true of Issidae. For example, *Hysteropterum albaceticum* was shown to have several bivalents easily distinguishable in meiotic cells by characteristic banding patterns (Kuznetsova et al. 2009). In the same paper, *Agalmatium bilobum* was shown to have C-bands on the largest and three medium-sized bivalents. Closely related species occasionally share the same or similar patterns as in *Mycterodus colossicus* and *Mycterodus* sp. (present study), both having telomeric C-bands on the largest and one of the medium-sized bivalents. On the other hand, *Falcidium doriae* and *Falcidius limbatus* were demonstrated to differ extensively in their C-band pattern (Kuznetsova et al. 2010). Some additional examples can be found in Kuznetsova et al. (2010). Based on the data obtained, it can be stated that the gain and loss of heterochromatin is an important source of karyotype diversification in the Issidae.

### Chromosomal mapping of repeated DNAs by fluorescence *in situ* hybridization (FISH)

Over the past decades, the FISH technique revolutionized the cytogenetic analysis providing significant advances on evolution of different insect groups with holokinetic chromosomes. At present, telomeres and the major rDNA loci are the most widely documented chromosomal regions in insects, including the order Hemiptera (e.g. Blackman et al. 2000, Manicardi et al. 2002, Monti et al. 2011a, b, Grozeva et al. 2011, 2014, Panzera et al. 2012, Chirino et al. 2013, Maryańska-Nadachowska et al. 2013, Pita et al. 2013, Bardella et al. 2013, Golub et al. 2014, 2015, Kuznetsova et al. 2012, 2015a). In addition, recent publications have shown that the number and chromosomal locations of the major rDNA multigene families are useful for the study of karyotype evolution in other insect groups (e.g. Grzywacz et al. 2011: Orthoptera; Gokhman et al. 2014: Hymenoptera; Vershinina et al. 2015: Lepidoptera; Lachowska-Cierlik et al. 2015: Mantophasmatodea; Mora et al. 2015: Coleoptera).

In Auchenorrhyncha, most cytogenetic studies were carried out by standard staining and conventional chromosome banding techniques. In this large hemipteran (= homopteran) group, FISH with rDNA and conserved insect telomeric (TTAGG)*_n_* repeats has so far been applied to 25 species, including 8 species of the genus *Philaenus* Stål, 1864 from the froghopper family Aphrophoridae (Maryańska-Nadachowska et al. 2013, Kuznetsova et al. 2015c); *Mapuchea chilensis* (Nielson, 1996) from the leafhopper family Myerslopiidae (Golub et al. 2014); 5 species of the genus *Alebra* Fieber, 1872 from the leafhopper family Cicadellidae (Kuznetsova et al. 2015b); and 11 species of the planthopper family Issidae (present paper). In addition, Frydrychová et al. (2004) reported on telomeric sequences in *Calligypona pellucida* (Fabricius, 1794) from the planthopper family Delphacidae. In all examined species, including those studied here, the presence of the (TTAGG)*_n_* telomeric repeat, known as the ancestral insect DNA motif of telomeres (Frydrychová et al. 2004), was detected.

The major rDNA loci were shown to vary in number (1 or 2 per haploid set) and chromosome location (autosomes, sex chromosomes or both; terminally or interstitially) in different species of Auchenorrhyncha. For example, in *Mapuchea chilensis* (2n = 16 +XY), the 18S rDNA clusters were present on a medium-sized pair of autosomes. In the karyotypically uniform genus *Alebra* (2n = 22 + X), they seem conserved and located on the largest pair of autosomes. In the genus *Philaenus*, which includes species with different chromosome numbers and karyotype structure, variation in number (1 or 2 per haploid set) and location (autosomes, sex chromosomes or both) of ribosomal genes was observed suggesting plasticity of the genomic organization within the genus. In the all species (11) of the Issidae from 8 genera and the three tribes, the 18S rDNA clusters were only detected in the largest autosomal pair. Basically, rDNA loci were located in an interstitial position, while in *Issus lauri* and *Zopherisca tendinosa* they were found at chromosomal ends suggesting that chromosomal rearrangements involving rDNA sequences occurred in the evolution of these unrelated species. In several karyotypes, FISH demonstrated size heteromorphism of rDNA clusters, suggesting that it can be attributed to differences in the number of ribosomal cistrons.

### A brief comparison between families of the “issidoid group”

Among the families Caliscelidae, Acanaloniidae, Tropiduchidae and Nogodinidae, which are phylogenetically related to the Issidae, data on karyotypes and the number of follicles are still very scarce (Kuznetsova et al. 1998, 2010, Maryańska-Nadachowska et al. 2006), while molecular cytogenetic data are not yet available.

### Follicle number

The “issidoid” families Caliscelidae, Acanaloniidae, Tropiduchidae and Nogodinidae taken together have currently only 11 species with known testis structure (Kuznetsova et al. 1998, Maryańska-Nadachowska et al. 2006). The relatively high and variable follicle numbers of the Issidae resemble the situation in the families Nogodinidae and Acanaloniidae, but not in the families Caliscelidae and Tropiduchidae, which share low and relatively stable numbers. In the four studied Nogodinidae species, numbers 5, 9 and 24 were observed, with the latter value found in two unrelated species, *Biolleyana pictifrons* Stål, 1864 and *Pisacha* sp. (Kuznetsova et al. 1998), whereas in the family Acanaloniidae, the only examined species, *Acanolonia bivittata* (Say, 1825), has 13 follicles per testis (Maryańska-Nadachowska et al. 2006). In the Tropiduchidae, the three studied species have either 6 or 3 follicles (Kuznetsova et al. 1998), while each of the three studied species of Caliscelidae has 6 follicles per testis (Maryańska-Nadachowska et al. 2006).

### Karyotype

The currently available data on the families Tropiduchidae, Nogodinidae, Caliscelidae and Acanaloniidae concern just 13 species (Kuznetsova et al. 1998, 2010, Maryańska-Nadachowska et al. 2006). The 2n = 26 + X and secondarily derived 2n = 24 + XY chromosome complements are shared by Issidae and Nogodinidae. In the latter family, *Bladina magnifrons* Walker, 1858 and *Biolleyana pictifrons* have 2n = 26 + X, whereas *Mindura subfasciata kotoshonis* Matsumura, 1941 and *Pisacha* sp. share 2n = 24 + XY (Kuznetsova et al. 1998). In the Tropiduchidae, Achilorma ?bicincta Spinola, 1838 was found to have 2n = 26 + X, whereas the three other studied species have different karyotypes, i.e., 2n = 24 + X in *Tambinia bizonata* (Matsumura, 1914) and *Barunoides albosignata* Distant, 1912, while 2n = 28 + X in *Varma distanti* Melichar, 1914 (Kuznetsova et al. 1998). Putative ancestral issid karyotype of 2n = 26 + X (Kuznetsova et al. 2010), has not yet been found in the families Caliscelidae and Acanaloniidae (Maryańska-Nadachowska et al. 2006).

### Concluding remarks

Based on the currently available data, which are still highly insufficient, we can infer that Issidae are characterized by 10 follicles per testis as the most frequent number, the presence of canonical insect telomeric repeats (TTAGG)*_n_*, a stable karyotype constitution with the predominant karyotype of 2n = 26 + X(0), and the major rRNA gene clusters located on the largest pair of autosomes. A much broader taxonomic coverage is necessary to discuss possible implications of the above characters for the taxonomy and phylogeny of the Issidae.
